# The Tri-State Medical Society

**Published:** 1894-10

**Authors:** 


### THE TRI-STATE MEDICAL SOCIETY.

This Society, representing the States of Georgia, Alabama, and Tennessee, will meet in Atlanta on the 9th, 10th, and 11th of this month. The place of meeting will be the ballroom of the Kimball House. A large attendance is very much desired. An interesting program of papers has been prepared, and several of our prominent Northern friends will be present as invited guests. Of late some objection has been made to the *name* of the Society, and the advisability of a change will be discussed. The physicians of Atlanta will try to make the sixth session of the Society pleasant, profitable and successful.

Partial list of papers:

Treatment of Stricture of the Urethra by Electrolysis, P. L. Brouillette, Huntsville, Ala.; Uterine Cancer, George R. West, Chattanooga; The Responsibility of a Class of Criminals from a Medico-Legal Point of View, John C. LeGrand, Anniston, Ala.; The Treatment of Stone in the Kidney, W. E. B. Davis, Birmingham, Ala.; The Treatment of Injuries and Inflammation of the Joints, Wm. L. Nolen, Chattanooga; Tuberculosis of the Kidney and Bladder, H. Berlin, Chattanooga; Some Remarks upon Brain Surgery, with Report of Cases, Paul F. Eve, Nashville; Pernicious or Inveterate Vomiting of Pregnancy—a Plea for the Mother, Based on Cases in Actual Practice, E. A. Cobleigh, Chattanooga; The Surgical Treatment of Empyema, J. A. Goggans, Alexander City, Ala.; Electro-Therapeutics, J. P. Stewart, Chattanooga; Headaches; Their Etiology and Treatment, R. P. Johnson, Chattanooga; Prognosis and Treatment of Placenta Previa, Richard Douglas, Nashville; Tuberculosis of the Nasal Bones; Report of a Case, B. F. Travis, Chattanooga; Appendicitis; Its Surgical Treatment, with Report of Cases, R. J. Trippe, Chattanooga; Puerperal Septicemia, with Cases Illustrating the Several Varieties, J. R. Rathmell, A.M., M.D., Chattanooga; Hygienic Treatment of Syphilis, T. M. Baird, Hot Springs, Ark.; The Pathological Import of Albumen in the Urine, E. B. Ward, Selma, Ala.; A Report of Some Rare Surgical Lesions Connected with the Liver, John A. Wyeth, New York; Is There Danger of not Getting Good Union after Tenotomy? C. W. Barrier, Columbus, Ga.; The Obstructive Urinary Diseases, W. L. Gahagan, Chattanooga; Reflex Neuroses in the Male, Andrew Boyd, Scottsboro, Ala.; The Induction of Labor to Prevent Blindness, Frank Trester Smith, Chattanooga; Notes on the Management of the Infant after Birth, Frank S. Parsons, Philadelphia; How to do Abdominal Section without Fuss, Feathers and Foolishness, and with Immunity from Sepsis, Joseph Price, Philadelphia; Treatment of Uterine Fibroids, W. Gill Wylie, New York; Outline of the History of Medicine and Surgery in Georgia, L. B. Grandy, Atlanta; Burns and the Treatment Thereof, T. Ellis Drewry, Griffin, Ga.; Some Causes that Lead to Invalidism in Women, J. B. S. Holmes, Atlanta; Essentials of Obstetric Nursing, R. R. Kime, Atlanta; The Treatment of Smallpox, C. H. Holland, Chattanooga; Amputation of the Mamma for Carcinoma and Treatment of the Axilla, B. W. Bizzell, Atlanta; Excision of Malignant Tumors of the Breast, Willis F. Westmoreland, Atlanta; Migraine; Its Etiology and Treatment, Hugh Hagan, Atlanta; The Use of Hydrastis Canadensis in Diseases of Mucous Membranes, P. R. Cortelyou, Marietta, Ga.; "Mixed Infection;" Report of Cases, M. B. Hutchins, Atlanta; "Paresis and Paralysis of the External Rectus of the Eye with Report of Two Cases," Dunbar Roy, Atlanta; "Combination of Carbolic Acid and Camphor as an Antiseptic and Local Anesthetic," William Perrin Nicolson, Atlanta; "Urethral Surgery Ten Years Ago and To-day," T. C. V. Barkley, Chattanooga; "Slaughter of the Innocents," E. van Goidtsnoven, Atlanta; "Some Points in Rectal Surgery," J. M. Mathews, Louisville; "Reform in the Treatment of the Neurotic and Insane Viewed from the Gynecological Standpoint," Charles A. L. Reed, Cincinnati; "Adenoids and their Sequelæ," Arthur G. Hobbs, Atlanta; "Unusual Nervous Phenomena in a Case of Fracture of the Fifth Cervical Vertebra, with its Pathology," W. C. Townes, Ph.B., M.D., Chattanooga; "Some Practical Points in the Treatment of Typhoid Fever," James B. Baird, Atlanta; "The Hygiene of the Hospitals and Prison Camps of the Georgia Penitentiary," W. O'Daniel, Atlanta; "Treatment of Typhoid Fever," John Eliot Woodbridge, Youngtown, O.; "Hypertrophic Rhinitis; Some of the Reflexes," George Brown, Atlanta.

---

HERMAN VON HELMHOLTZ, the distinguished physicist and physiologist, died September 8th, aged seventy-three years. He had been for years one of the foremost scientific men in Europe, and it is impossible to estimate fully the extent to which his researches had enriched the physical sciences. For twenty years he was professor of physiology in different German universities, and during this period (1851) his observations on the course of light rays in the eye led him to devise the ophthalmoscope. He also invented the ophthalmometer, and by it discovered the mechanism of accommodation. In 1855 he published a "Hand-Book of Physiological Optics," which laid the foundation of modern ophthalmology. In 1871 Helmholtz became Professor of Physics in the University of Berlin. In this place he conducted an immense amount of investigation in the phenomena of electricity, heat, light, and hydrodynamics. He wrote numerous essays relating to atmospheric currents, the cosmic theory, the conservation of energy, etc. He was also the author of several popular lectures on scientific subjects. Three years ago his friends gave him a complimentary ovation on the occasion of his seventieth birthday. His life had been one of great intellectual labor, and every subject he undertook was benefited by his accurate and patient investigation.

